# Virtual Reality Exposure Therapy for Anxiety Disorders: Small Samples and No Controls?

**DOI:** 10.3389/fpsyg.2016.00326

**Published:** 2016-03-11

**Authors:** Sarah Page, Matthew Coxon

**Affiliations:** Faculty of Health and Life Sciences, York St. John UniversityYork, UK

**Keywords:** virtual reality, anxiety, post-traumatic stress disorder, sample size, control, exposure therapy

Virtual Reality Exposure Therapy (VRET) has the potential to help clinicians manage a range of symptoms related to anxiety disorders (e.g., Rothbaum et al., 1995; North et al., 1996). On a theoretical level the proposed underlying mechanisms reflect those in traditional exposure therapy; emotional processing is facilitated by activating the underlying fear structure through confrontation with the feared stimuli, allowing responses to be modified in a controlled therapeutic setting, so the stimuli will become less anxiety provoking when subsequently perceived (Rothbaum et al., 2000). In a VRET treatment protocol, an individual is immersed into a virtual environment that allows for sensory exposure to the feared stimuli via computer-generated displays. It permits the individual to face their triggers in a safe environment and allows the therapist to control the intensity and duration of the stimuli, based on their clinical appraisal. The environments can be tailored to represent the individual's fears and, in the case of Post-traumatic Stress Disorder (PTSD), can be used to recreate a traumatic experience (e.g., Roy et al., 2006; Rizzo et al., 2009). VRET is usually delivered via a head-mounted display which tracks the users' head-movements and allows for real-time updating of the scenes they can see (Wiederhold and Wiederhold, 2005).

The use of VRET for clinical purposes has been investigated empirically for the last 20 years (e.g., Williford et al., 1993; Lamson and Meisner, 1994; Rothbaum et al., 1995). Over this period the evidence base has grown substantially and multiple anxiety-related clinical diagnoses have been investigated, including acrophobia (e.g., Rothbaum et al., 1995; Emmelkamp et al., 2002), aviophobia (e.g., Rothbaum et al., 2000; Mühlberger et al., 2006), arachnophobia (e.g., Garcia-Palacios et al., 2002; Cote and Bouchard, 2005; Bouchard et al., 2006), and PTSD (e.g., Rothbaum et al., 1999; Ready et al., 2006). The effectiveness of VRET is now well-established: four independent meta-analyses have concluded that such interventions lead to significant decreases in anxiety-related symptoms (Parsons and Rizzo, 2008; Powers and Emmelkamp, 2008; Opriş et al., 2012; Morina et al., 2015). Whilst the clinical efficacy of VRET is well supported, there continues to be the perception that the strength of the evidence base is weakened by three key methodological limitations: the use of small sample sizes (e.g., McLay et al., 2014; Castro et al., 2014; Morina et al., 2015); a lack of appropriate control groups (e.g., Nelson, 2012; McCann et al., 2014); and, more broadly, a lack of randomized controlled trials (RCTs: e.g., Nelson, 2012; McCann et al., 2014). These suggestions are by no means new and have been highlighted repeatedly across the years. It is generally portrayed that this picture has not changed as the research literature has grown (e.g., McCann et al., 2014), but to what extent is that true for these common concerns?

There are several different ways in which the above question could be answered. Here we adopted a systematic quantitative approach and inspected two discrete sets of data. Firstly we inspected all of the studies included in Parsons and Rizzo's (2008) original meta-analysis to obtain a picture of the research published in the 10 years following the seminal study by Rothbaum et al. (1995; for full details of search and inclusion criteria, see Parsons and Rizzo, 2008, pp 3). This consisted of 22 studies, published between 1995 and July 2006 and will be referred to here as the “early” studies. The same criteria were used to identify research literature for our second dataset, those published between August 2006 and June 2015. This yielded a further 49 studies and will be referred to here as the “later” studies. All 71 papers were reviewed to determine how they perform in relation to the most common concerns raised, specifically whether they used a small sample (specified as *p* < 30, in line with Parsons and Rizzo, 2008), whether they included a control condition, and whether an RCT design was used.

A surface inspection of the papers appears to be broadly consistent with the perceptions outlined above. The proportion of studies with small sample sizes has increased within the “later” studies (53%) compared to the “early” studies (41%). Small sample sizes are more common than they used to be. In a similarly discouraging outcome, a smaller proportion of the “later” studies (69%) make use of control groups compared to the early studies (82%). Control groups are used less often than they used to be. The use of RCT designs has remained relatively stable across time with 64% of the early studies using such designs, and 63% of the later studies using them. Given this picture it is not unreasonable that researchers continue to raise these issues. If anything, the proportion of studies with these methodological issues is increasing rather than decreasing. However, this pattern is not consistent across the literature as a whole and a closer inspection of the data across different anxiety disorders reveals a different picture.

On scrutiny of individual anxiety disorders, it is clear research in some areas has significantly progressed toward overcoming the methodological concerns presented. For example, in early studies into arachnophobia only 20% used a sample size of 30 or more, 60% used a control group and 67% conducted RCTs, however 100% of later studies met these criteria. Similarly, none of the early studies into public speaking used a sample size greater than 30 and only 50% used a control group or conducted an RCT. 100% of later studies into public speaking used a sample size above 30, 84% used a control group and 83% of studies were RCTs. It is apparent that research into arachnophobia and public speaking has improved considerably, with small sample sizes no longer an issue and an increasing number of studies using a control condition and RCT design.

The picture when looking at other clinical disorders varies slightly more. Studies into aviophobia out-perform the literature as a whole: 85% include a sample size greater than 30, a control group and an RCT design. However, the methods have, over time, followed the same trend on all three methodological issues, dropping from 100% in early studies to 75% in later studies. Research into aviophobia therefore includes an increasing number of studies that do not use a sufficient sample size, a control group or an RCT design. In contrast, research into acrophobia has an inconsistent pattern. Overall, the studies perform better than average in terms of the percentage of studies using more than 30 participants and the percentage of studies using a control group (71 and 86%, respectively), yet performance has deteriorated over time, from 75 to 66% for sample size and 100–66% for use of control group. In a contrasting finding, the use of RCTs has improved over time, increasing from 50 to 67%. Studies into acrophobia therefore make use of small sample sizes and neglect to use a control group. In contrast, the use of an RCT design has increased; the percentage of studies utilizing this design is now approximately in line with the overall literature. Studies into agoraphobia present a similarly complex pattern. All early studies used a control group, an RCT design and a sample size greater than 30. The performance of the later studies is strikingly varied, 100% used a control group, 90% were RCTs and only 50% used a sample size greater than 30. Research into agoraphobia supports the conception that studies are still being conducted using small sample sizes. It is evident research into individual clinical disorders does not uniformly fit the overall trends in the literature, providing mixed support for the concerns highlighted.

Compared to research into the use of VRET to treat other clinical disorders, studies focusing on agoraphobia show a marked decrease in performance on the particular concern of sample size. There are a number of possible reasons for this particular limitation. The high drop-out rate of participants means researchers have to recruit a higher number of participants to have sufficient completers, for example Peñate et al. (2008) recruited more than 30 participants but experienced a 22% drop-out rate leaving them with less than 30 participants. They cited lack of motivation, non-agreement with therapy and treatment failing to meet participant expectations as the reasons (Peñate et al., 2008). Of the studies included, drop-out rate is as high as 37.5% (Castro et al., 2014), with further problems being cited as participants feeling that the exposure made them “suffer,” participants having many previously failed attempts at treatment and the particularly high severity of symptoms (Pelissolo et al., 2012; Castro et al., 2014). It has been highlighted that the clinical symptomology in agoraphobia is more complex than in specific phobias, impacting recruitment and attrition rates (Botella et al., 2007; Malbos et al., 2012).

Finally, compared to research in other clinical areas it appears that the literature on using VRET for PTSD contains a particularly high number of studies with smaller sample sizes and are less likely to use a control group or RCT design. In the past 10 years there has been a substantial increase in the proportion of the literature concentrating on treating PTSD, increasing from 4.5% of the “early” studies (*n* = 1) to 22% of the later studies (*n* = 11). Only 8% of all studies regarding PTSD used a sample size greater than 30, compared to 59% of studies into other anxiety disorders. 25% of the studies into PTSD use a control group and 25% use an RCT design, whereas 83 and 71% of the other studies fulfill these criteria, respectively. It is evident that research concentrating on PTSD does not consistently meet the methodological criteria that have been consistently raised as concerns.

There are a number of possible explanations why research concerning PTSD features these methodological limitations more than others. The lower prevalence of individuals developing PTSD than specific phobias (Kessler et al., 2012) means a smaller population of potential participants for studies. Furthermore, of the studies into PTSD, 83% used a military population; specific problems with recruitment have been highlighted within this population (Brown and Bruce, 2015). McLay et al. (2011) suggested stigma around mental health, constant changes to work schedules and changes to military personnel overseeing the participants as barriers to recruitment. This difficulty in recruiting participants therefore makes it problematic to include a control group and still be able to report reliable findings (Ready et al., 2010).

## Conclusions

There has been a common conception that studies using VRET to treat anxiety disorders continue to use small sample sizes and lack appropriate control groups or an RCT design: these concerns have been recently and repeatedly raised, suggesting some researchers do not consider the situation to have improved. In a preliminary survey of the literature it appeared these frequently expressed concerns are verified, with a decrease in use of larger sample sizes and control groups, and the proportion of studies using an RCT design unchanged since 2006. However, the underlying patterns differ considerably according to the clinical disorder investigated, making it important that findings from the literature are not dismissed as a result of the methodological limitations highlighted. Research concerning both arachnophobia and fear of public speaking has all but eliminated these concerns. The pattern is more mixed for studies into agoraphobia, acrophobia, and aviophobia, with research concerned with PTSD rarely meeting the criteria.

Larger samples, of course, allow for more precise estimates of the treatment's effect, and more precise estimates of confidence intervals, leading to more meaningful comparison with alternative treatment options. Moreover, the use of appropriate control conditions and RCT designs enables researchers to compare VRET to other treatment options with more confidence and more readily illustrates its clinical effectiveness to practitioners. These are not minor issues and are of importance to both researchers and practitioners in assessing the strength of the evidence base. However, the common conception is that VRET research is relatively weak on these issues. It is recommended here that both researchers and practitioners take a more differentiated view of the literature according to the anxiety disorder of interest. The evidence base has grown sufficiently now to begin to make such judgements.

## Author contributions

SP contributed by developing the concept, interpretation of research, drafting, revising, and finalizing the transcript. MC contributed by assisting with developing the concept, revising the transcript, and approving the final version to be published. SP and MC agree to be accountable for all aspects of the work in ensuring that questions related to the accuracy or integrity of any part of the work are appropriately investigated and resolved.

## Funding

The preparation of this article was supported by the SPARK Postgraduate Employability & Enterprise Scholarship provided by York St John University.

### Conflict of interest statement

The authors declare that the research was conducted in the absence of any commercial or financial relationships that could be construed as a potential conflict of interest.
